# Subtle concentration changes in zinc hold the key to fibrillation of α-synuclein: an updated insight on the micronutrient’s role in prevention of neurodegenerative disorders

**DOI:** 10.3389/fmolb.2025.1603364

**Published:** 2025-07-10

**Authors:** Samudra Prosad Banik, Debasis Bagchi, Pradipta Banerjee, Sanjoy Chakraborty, Manashi Bagchi, Chaitali Bose, Debasmita De, Sreemoyee Saha, Sudipta Chakraborty

**Affiliations:** ^1^ Department of Microbiology, Government General Degree College, Narayangarh, Rathipur, West Bengal, India; ^2^ Department of Biology, College of Arts and Sciences, Adelphi University, Garden City, NY, United States; ^3^ Department of Psychology, Gordon F. Derner School of Psychology, College of Arts and Sciences, Adelphi University, Garden City, NY, United States; ^4^ Department of Pharmaceutical Sciences, College of Pharmacy and Health Sciences, Texas Southern University, Houston, TX, United States; ^5^ Department of Surgery, University of Pittsburgh, Pittsburgh, PA, United States; ^6^ Department of Biological Sciences, New York City College of Technology/CUNY5113, Brooklyn, NY, United States; ^7^ Department of R&D, Dr. Herbs LLC, Concord, CA, United States; ^8^ Department of Nutrition, Government General Degree College, Narayangarh, Rathipur, West Bengal, India; ^9^ Department of Microbiology, Maulana Azad College, Kolkata, West Bengal, India

**Keywords:** neurodegenerative disorders, α-synuclein, synucleinopathies, Parkinson’s disease, liquid-liquid phase separation, zinc as micronutrient, ZnO nanoparticle

## Abstract

Misfolded proteins have been found to be at the core of an increasing number of cognitive ailments. α-synuclein, a resident chaperone of the neurosynaptic cleft has been implicated in a major share of these neurodegenerative diseases. Over the years, a daunting task for researchers has been the identification of the complex set of conditions which govern the Substantia nigra microenvironment for transformation of α-synuclein from a functional and grossly structureless chaperone to toxic cross-β fibrils. An abundance of Reactive Oxygen Species and a drop in pH of the solvent have been identified to be the key drivers of the fibrillation process which is initiated by Liquid-Liquid phase separation of α-synuclein droplets. Zinc is a significant micronutrient of the human body integral to the proper functioning of the nervous system as well as holistic cognitive development. Many recent studies have deciphered that metal ions including zinc facilitate the fibrillation of α-synuclein by shielding negative charges at the C terminus of the protein. Zinc preferentially binds to Asp121 at the C terminus and His50 at the N terminus to promote fibrillation. On the contrary, zinc has many protective roles to retard fibrillation of the protein at the same time. It downregulates ROS and assists chaperones which prevent non-native aggregation of α-synuclein. The ability of zinc to bind preferentially to α-synuclein coupled with the advent of ultrasensitive detection technologies such as the Surface Enhanced Raman Spectroscopy has led to the prospects of zinc-oxide nanoparticles as effective tools to probe the α-synuclein-based biomarker for early detection of protein aggregates in the body fluid. This review summarizes the significant mechanistic findings which has facilitated our understanding of the fibrillation of α-synuclein, the precise role and mechanism of zinc involved therein and the prospects of using zinc in designing efficient tools for diagnosis of Parkinson’s Disease and other synucleinopathies.

## 1 Introduction

Amyloids are non-native aggregates formed from cytosolic or membrane associated proteins in neurons which have defined physiological functions but transform into soluble and toxic fibrillar entities under poorly understood altered conditions. Amyloid aggregation is considered to be one of leading causes behind cognitive impairment and onset of several debilitating neurodegenerative diseases (Wells et al., 2021; Wolfe and Cyr, 2011) collectively referred to as amyloidosis (Metkar et al., 2024) Tau proteins representing the characteristic pathology of Alzheimer’s disease (Chen and Yu, 2023) are normally associated with stabilization of neuronal microtubules (Jesu et al., 2004), but give rise to fibrillar tangles along with β-amyloid under a hyperphosphorylated state. Amyloid β protein, on the other hand, is implicated in regulation of synaptic function, improving memory and protection of neurons from oxidative stress (Bishop and Robinson, 2004).

Probably the most significant form of amyloid related neuropathy is associated with α-synuclein, a major neurosynaptic chaperone which mediates release of neurotransmitters (Sulzer and Edwards, 2019). The protein owes its name to its simultaneous localisation in both synaptic vesicle as well as nuclear envelope in a type of stingray (Clayton and George, 1998). In addition to its role as a synaptic chaperone, α-synuclein is also indirectly associated with the regulation of activity of glutamatergic ionotropic and metabotropic receptors activity by virtue of its interaction with other proteins (Calabresi et al., 2023). α-synuclein, one of the major causes of amyloidosis has also been implicated in the highest known numbers of cognitive ailments including Parkinson’s disease, Lewy Body Dementia, Multiple System Atrophy and many other cognitive disorders collective known as α-synucleinopathies (Calabresi et al., 2023). It is a 140-residue protein with three distinct domains; a positively charged N-terminal region (residues 1–60), a hydrophobic non amyloid β component (NAC) region (residues 61–95), and a C-terminal region with amino acids bearing a net negative charge (residues 96–140) (Ohgita et al., 2022). The presence of the two signature features of an aggregation prone protein, namely a hydrophobic region and a sequence with a strong propensity to adopt a beta pleated sheet structure make α-synuclein an ideal candidate for fibrillation; however, the negative charge at the C terminus and its long range non-covalent interaction with the N terminal domain protects the protein from losing its native state and committing to fibrillation. Fibrillation of α-synuclein leads to the formation of a cross beta pleated sheet which causes cellular toxicity by creating pores in the membrane, inducing misfolding of other proteins and eliciting other signalling pathways which results in loss of cellular homeostasis. In spite of a plethora of concerted efforts to understand the molecular basis of fibrillation (Brás and Outeiro, 2021), scientists are yet to decipher the combination of responsible intrinsic and/or extrinsic factors (Longhena et al., 2020; Srinivasan et al., 2021). The available scientific evidences are all equivocal of the fact that synuclein fibrillation consists of two distinct steps a) Nucleation or initiation of fibrillation and b) elongation of fibrillation. Secondary nucleation pathways from dissociated protofibrils are also a prominent part of the process thus resembling a prion like propagation of these generally toxic entities (Coles et al., 2025).

Since the major guiding factor of α-synuclein fibrillation is its charge dependency, bivalent metal ions also play a significant role in governing fibrillation kinetics (Atarod et al., 2022). Particularly, metal ions are believed to mimic the effect of pH by neutralizing the negative charge of the C-terminus, thus increasing the hydrophobicity of the central domain with simultaneous propensity to switch over to the beta pleated sheet (Byrd et al., 2023).

Zinc is a significant trace element with myriad physiological and biochemical functions essential for life. It plays a key part in boosting neuronal health and the micronutrient’s deficiency has been associated with oxidative stress, programmed cell death and necrosis of neurons eventualizing into a plethora of neurodegenerative disorders (Vali et al., 2025). Zinc’s role in Parkinson Disease pathology, in particular, has been intriguing to understand since high zinc concentration promotes fibrillation of α-synuclein (Gao et al., 2022) whereas lower levels assist the cellular chaperones in preventing fibrillation (Al-Harthi et al., 2022). The selective binding of zinc to key regions of α-synuclein has also opened up new avenues for the development of technologies to probe α-synuclein based biosensors for early detection of neuropathies.

The diverse facets of α-synuclein fibrillation render it practically inconceivable to predict the set of extrinsic and intrinsic factors responsible for its phase transition. Metal ions present an additional level of complexity in this regard owing to their differential effect in modulating the fibrillation potential. The primary objective of this review is to present a comprehensive insight of the effect of metal ions, particularly zinc, in modulating α-synuclein aggregation, fibrillation and resultant toxicity analyses along with the technological and scientific challenges involved therein. In addition, the exciting new frontiers of amyloid research, namely, the role of *E. coli* amyloid curli in shaping the amyloidogenic behaviour of cellular proteins via the gut-brain axis, and the molecular connection between diabetes and neurodegenerative disorders mediated by advanced glycated end products have also been discussed in the perspective of the role of zinc involved therein.

## 2 α- Synuclein as the chief mediator of synucleinopathies

Parkinson’s disease (PD) is the second most abundant neurological disease after Alzheimer which primarily affects co-ordination in movement. The disease, which was described as Shaking Palsy by its discoverer, James Parkinson in the year 1817 (Goetz, 2011), is caused primarily by death of the dopamine neurons in the Substantia nigra (SN), a region named after the high levels of melanin present in the dopamine neurons in unaffected individuals (Zecca et al., 2003). Loss of dopaminergic neurons results in gross cardinal motor dysfunctions leading to bradykinesia, muscle stiffness, tremors in resting posture, as well as postural and gait impairment (Ramesh and Arachchige, 2023), features which are the hallmarks of PD. Lewy Body Dementia (DLB) is another frequently occurring dementia involving progressive memory loss and hallucinations with cortical depositions of insoluble α-synuclein (Ye et al., 2020). Yet another example of α-synuclein associated cognitive ailment is Multiple System Atrophy which involves depositions of Glial Cytoplasmic Inclusions (GCI) formed of hyperphosphorylated α-synuclein inclusions in oligodendrocytes, better known in the disease perspective as Papp-Lantos bodies (Jellinger, 2014). The characteristic features of MSA include both motor and autonomic disturbances including Parkinsonism, cerebellar ataxia, orthostatic hypotension, urinary dysfunction and cardiovascular issues which often has fatal consequences (Campese et al., 2021). Together, the spectrum of α-synuclein associated neuropathies are referred to as synucleinopathies.

Most of the PD cases are linked to late adult onset and are sporadic in nature. However, in 1997, the first gene associated with familial PD was identified which coded for the presynaptic chaperone protein called α-synuclein (Nussbaum, 2017). The pathology associated with mutant α-synuclein was first detected in the form of characteristic black dots surrounded by vaguely defined halos termed Lewy bodies in histopathological sections of neuronal soma of patient brains affected with this rare inherited form of the disease. Later on, the molecular genetic basis of the disease was linked to a transition mutation in the snca gene A53T which results in the substitution of alanine to threonine in the N terminal 53rd amino acid of the protein leading to its abnormal aggregation (fibrillation) (Lee and Trojanowski, 2006). Subsequently many other mutations were found to be associated with familial forms of PD (Flagmeier et al., 2016) including the A30P mutant aggravating PD by reduced autophagic clearance of protein aggregates (Lei et al., 2019) and E46K mutant which promotes fibrillation of N acetylated α-synuclein by abolishing the long-range electrostatic interactions (Zhao et al., 2020). Lewy bodies are clumps of misfolded/non-native protein aggregates named after their discoverer Frederick Lewy in 1912 (Mueller et al., 2017). Apart from α-synuclein, Lewy bodies also contain many other aggregated cellular proteins, which together constitute an interactome to promote fibrillation of α-synuclein (Lashuel and Novello, 2021). Subsequent clinical cases revealed that α-synuclein pathology is not only restricted to the soma but also extends to neurites which led to the coinage of the term “Lewy neurites” (Braak et al., 1999).

Under normal physiological conditions α-synuclein exists mostly in an N acetylated membrane bound form which results in adoption of an α-helical structure (Runfola et al., 2020). This membrane bound conformation effectively prevents its fibril formation and subsequent toxic consequences (Bell et al., 2022). However, without the acetylation, it is naturally an Intrinsically Disordered Protein lacking any discernible secondary structure (Zhang et al., 2020). Point mutations or gross genetic changes have been known to induce the abnormal conformational changeover of α-synuclein (Waxman and Giasson, 2009). This and many other hitherto uncharacterised factors induce accumulation of pathogenic inclusions differently termed as Lewy bodies in neurons of PD patients (Marotta et al., 2021) or glial cytoplasmic inclusions in oligodendrocytes of Multiple System Atrophy (MSA) patients (Reddy and Dieriks, 2022). Apart from mutations, other significant factors accounting for α-synuclein fibrillation are changes in pH of the microenvironment (Frey et al., 2024), presence of other metal ions temperature (Ariesandi et al., 2013), oxidative stress (Puspita et al., 2017), phosphorylation (Ghanem et al., 2022; Kawahata et al., 2022), molecular crowding (Shtilerman et al., 2002) as well as presence of other proteins in the vicinity (Uversky et al., 2002). Deletion of a stretch of acidic residues along with proline in the C terminal region makes the protein more vulnerable to toxic fibril formation (Farzadfard et al., 2022a) due to loss of long-range electrostatic interactions with the N terminus. The proline residues in the C terminus plausibly function to bring the interacting residues in close vicinity. A particular point mutant of the protein, A53T, associated with familial PD is also known to fibrillate at a significantly higher rate than the normal protein (Coskuner and Wise-Scira, 2013) owing to its increased propensity to form beta fibrils as well as by partially negating the effect of long-range interactions between the N and C terminus. Effect of net charge of the protein on fibrillation is also explained by the observation that pH of the microenvironment is elevated as fibrillation proceeds, along with rise in pK_a_ of the acidic amino acids, in order to prevent association of N and C termini which might block the fibrillation process (Pálmadóttir et al., 2021).

The most acceptable theory to explain the departure of the native α-synuclein into the fibrillation pathway is the Liquid-Liquid phase separation (Ray et al., 2020). This is subsequently converted into a hydrogel-like entity consisting of amyloid oligomers and protofibrils (Mukherjee et al., 2023). The holistic effect of all extrinsic factors accounting for the phase transformation is to shield or neutralize the charges at the N or C terminal ends, as this has been also confirmed by specific site directed mutations of key residues of the protein (Pancoe et al., 2022).

### 2.1 Liquid-liquid phase separation drives α-synuclein fibrillation

**FIGURE 1:**
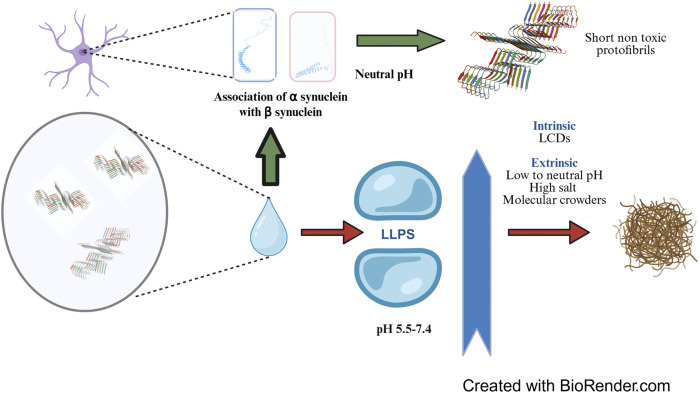
Liquid-Liquid Phase Separation (LLPS) of α-synuclein: LLPS and subsequent fibrillation of α-synuclein is promoted by intrinsic factors such as Low Complexity Domains and solution conditions including low to neutral pH, high salt concentration and presence of molecular crowding agents. LLPS results in local build-up in α-synuclein concentration to as high as 30-40 mM from the physiological concentration of around 50 µM which eventually causes formation of neurofibrillary tangles. In healthy brain, β-synuclein, a homologue of α-synuclein associates with the later at neutral pH and prevents the formation of long, toxic and fibrillar α-synuclein aggregates. The concepts and mechanisms depicted in the figure have been adopted from multiple studies as cited in the text. The protein structure cartoon shown in the figure has been downloaded from RSCB PDB (PDB ID 6OSM, Ni et al. 2019). Figure has been created with BioRender.

Liquid-liquid phase separation (LLPS) is a physical phenomenon which involves separation of a uniform solution of macromolecules into two distinct liquid phases with different densities of macromolecules, resulting in the formation of coacervate like structures. Phase separation is preceded by accumulation of multiple conformations of α-synuclein monomers some of which are structurally more adapted to develop into protofibrils (Dedmon et al., 2005). Brodie and co-workers used a combination of Molecular Dynamics simulation data substantiated with cross-linking mass spectrometry (MS) and single molecule Förster resonance energy transfer (smFRET) data to generate an ensemble of conformational spaces of such α-synuclein monomers (Brodie et al., 2019). The tendency of α-synuclein to undergo LLPS is facilitated by the presence of low complexity domains (LCDs), one or more sequence stretches with higher abundance of certain amino acids instead of the complete set of 20 amino acids (Mahapatra and Newberry, 2024). These sequences are mostly unstructured representing the IDR regions of proteins and initiate the formation of unique condensates where many molecules of α-synuclein slowly transform into the beta-pleated structure mediated by transient multivalent interactions. This results into a local increase in density of these proteins and marks the initiation of nucleation of the fibres. The process is facilitated by the presence of Polyethylene Glycol, a molecular crowding agent which brings α-synuclein molecules in close proximity by displacing water from the immediate vicinity for enhanced hydrogen bonding, non-covalent interactions and hydrophobic interactions (De Luca et al., 2025). Eventually, further α-synuclein molecules rope in and fibrils are extended leading to the formation of amyloid like hydrogel entities (Mukherjee et al., 2023) (Figure 1). The concentration of α-synuclein in these condensates can reach to as high as 30–40 mM (Dada et al., 2023) which leads to the formation of neurofibrillary tangles or the so-called Lewy bodies. Phase separation of α-synuclein under both cellular and *in vitro* conditions is guided by the Vesicle Associated Membrane Protein 2 (VAMP2) and its interaction with R-SNARE (Agarwal et al., 2024). The disease pathology is subsequently spread by prion like self-propagation of α-synuclein fibrils (Jan et al., 2021). The indispensable role of the C terminal domain in preventing fibrillation has been demonstrated by an engineered α-synuclein with truncated C terminus which not only displayed accelerated fibrillation kinetics but also promoted the phase separation of WT α-synuclein (Huang et al., 2022). Apart from the intrinsic sequence predisposition and presence of molecular crowding agents, many other extrinsic factors act as mediators of the LLPS; the more significant of these being the pH and the concentration of metal ions in the local microenvironment. At a pH of 7.4, primary nucleation proceeds spontaneously and is the fastest in the absence of other governing factors (Dada et al., 2023) whereas the critical concentration of α-synuclein needed to undergo LLPS is the least at pH 5.5 probably owing to the proximity of the isoelectric point of α-synuclein near that pH assisting separation of proteins from the solution (Hou et al., 2023). A high salt concentration in the medium promotes fibrillation and lowers down the critical concentration of the protein to undergo LLPS by neutralizing the charges at the N and C termini and promoting aggregation of the NAC region (Sawner et al., 2021). In addition to the charge neutralization, it also promotes effective salting out of the protein to induce phase separation.

Under physiological conditions, LLPS of α-synuclein is prevented by β-synuclein, a related protein of the synuclein family which is present at the presynaptic terminals at a similar concentration as that of α-synuclein (around 50 µM). Several independent reports have indicated that β-synuclein exerts its action by associating with α-synuclein and subsequently a) elongating the lag phase of fibrillation (Jain et al., 2018) and b) arresting secondary nucleation (Brown et al., 2016). β-synuclein has a 78% sequence identity with α-synuclein, differs in few critical aspects from the later including a 11 residue shorter NAC region, absence of cysteine and tryptophan residues and an abundance of proline and acidic residues and forms ordered structure more readily than α-synuclein (Hayashi and Carver, 2022). A higher number of proline residues is probably needed by the protein to make up for the folding flexibility curtailed by the shorter NAC region. A particular point mutant of β-synuclein, P123H, results in pathogenic deposits of Lewy Body Dementia (Psol et al., 2021) Li et al. found that β-synuclein associated with α-synuclein employing electrostatic interactions. In an *in vitro* microenvironment mimicking presynaptic terminal with equimolar amounts of α and β synuclein, the total mean surface area of α/β-Syn condensates was higher than that of α-Syn alone. Although the association of α/β-Syn condensates apparently promoted LLPS of α-synuclein, its corresponding nucleation and fibrillation was effectively prevented (Li et al., 2024). The pivotal role of NAC region in driving the aggregation is also supported by the observation that the second identified pathogenic point mutation V70M (Valine to Methionine) causes predisposition to a sporadic Lewy Body Dementia. Methionine is less hydrophobic than valine and therefore substitution to methionine should have lessened the aggregation propensity; therefore, this observation is probably a testimonial to the indispensability of Valine at the 70th position for NAC mediated aggregation of β-synuclein and its simultaneous association with α-synuclein.

### 2.2 Major physiological changes to account for the disease pathology of α-synuclein

Misfolding of α-synuclein has been linked to a plethora of cognitive ailments arising from the death of dopaminergic neurons and collectively referred to as synucleinopathies. The soluble protofibrils formed from α-synuclein exert their cytotoxicity through disruption of mitochondrial function, promotion of autophagy, lysosomal impairment and loss in regulation of calcium homeostasis (Calabresi et al., 2023). Particularly, the protein has been found to be associated with increased production of Reactive Oxygen Species (ROS) and elicitation of inflammatory response (Van Laar et al., 2020), the two primary reasons accounting for loss in cellular homeostasis.

### 2.3 Augmentation of ROS by α-synuclein and *vice versa*

**FIGURE 2:**
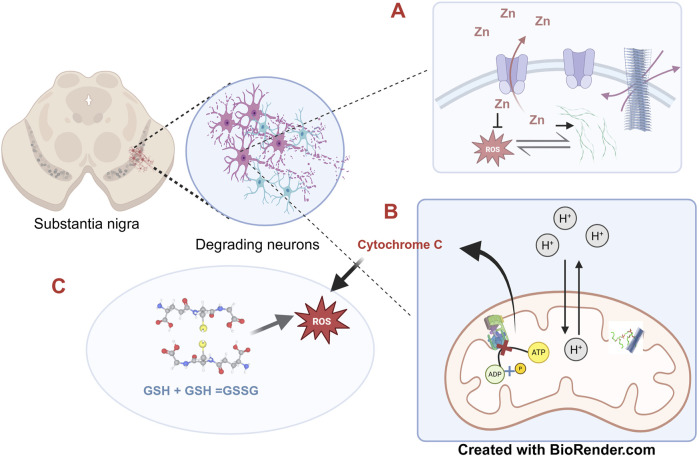
The multiple pathologies of α-synuclein-The substantia nigra in the basal ganglia of the mid brain represents one of the prime sites of α-synuclein mediated pathology where the misfolded protein accounts for the degeneration of the dopaminergic neurons. **(A)** The synaptic vesicles dissipate the otherwise toxic accumulation of excess zinc ions by the membrane associated ATPase, ATP13A2. A build-up of zinc promotes fibrillation of α-synuclein; however, at the same time also reduces ROS. α-synuclein and ROS augment each other. **(B)** Neuronal degeneration is accelerated by ATP depletion. Mitochondrial membrane permeability is altered by the toxic cross-beta α-synuclein fibrils through interaction with Cardiolipin, a major mitochondrial membrane lipid, which results in uncoupling of ATP synthesis with simultaneous release of cytochrome c. **(C)** α-synuclein fibrillation induced oxidative stress leads to accumulation of oxidised glutathione which in turn augments ROS thus self-perpetuating the toxicity. The concepts and mechanisms depicted in the figure have been adopted from multiple studies as cited in the text. Figure has been created with BioRender.

As stated earlier, α-synuclein fibrillates by addition of monomers after the initial nucleation; during elongation, smaller fragments of oligomers and protofibrils often gets detached from the primary fibril serving as additional points of nucleation thus aggravating the problem. This avalanche of soluble protofibrils is often believed to be more toxic than the stable full length cross-beta fibrils owing to their membrane permeabilization capability and interaction with various cellular organelles (Winner et al., 2011). Mitochondria represent the most vulnerable site of α-synuclein attack primarily owing to the latter’s interaction with cardiolipin, a major mitochondrial phospholipid with simultaneous formation of transient pores in the membrane (Ghio et al., 2019). The first case of α-synuclein independent PD case linked with mitochondria was reported with respect to consumption of banned drugs contaminated with 1-methyl-4-phenyl-1,2,3,6-tetrabydropyridine (MPTP). The toxic bioactive form of this compound 1-methyl-4-phenylpyridinium (MPP+) blocks the functioning of mitochondrial Complex-1 and NADH-ubiquinone oxidoreductase enzyme resulting in release of free electrons with concomitant ROS generation (Javitch et al., 1985). Creation of transient pores by α-synuclein oligomers induce similar effects of uncoupling electron transport from ATP production, resulting in release of free electrons and cytochrome c and concomitant ROS generation. Apart from directly modulating mitochondrial physiology, α-synuclein oligomers also amplify cytosolic peroxide flux resulting in oxidation of glutathiones (Van Laar et al., 2020). A logical explanation behind this observation comes from the fact that elongation of the polymeric fibrillar structure of α-synuclein requires a constant supply of free protons from the solution in order to mask the C terminal negative charges and prevent the interference of the N and C terminal association during fibrillation. In addition, α-synuclein oligomers can also elicit ER stress and consequent induction of Unfolded Protein Response pathway (Puspita et al., 2017). The consequent rise in intracellular calcium levels leads to further augmentation of ROS induced cellular damage. The diverse mechanisms by which misfolded α-synuclein inflict its cellular toxicity has been summarized in Figure 2.

In the reverse perspective, production of cellular ROS is also associated with fibrillation of α-synuclein (Esteves et al., 2008). In a superoxide dismutase 2 (SOD2) transgenic mouse model, development of synucleinopathies was significantly earlier as compared to the control mouse model which proved that a compromise in the capacity to quench free radical led to enhanced fibrillation of α-synuclein (Scudamore and Ciossek, 2018). In related studies, cytochrome c and peroxide radicals also resulted in crosslinking of α-synuclein monomers through the formation 3,3′-dityrosine linkages (Hashimoto et al., 1999; Ruf et al., 2008). Such crosslinking can arrest the formation of conventional cross beta fibrils but at the same time promote off-pathway aggregates as oligomers which are potentially more toxic.

### 2.4 Effect of pH on α-synuclein fibrillation

Aggregate formation and fibrillation of α-synuclein is favoured at slightly acidic pH, much like the endosomes and the lysosomes. This is explained by the fact that at neutral pH, the protein bears a net negative charge which is nullified on lowering the pH. The C terminal also gets fully protonated and uncharged at this pH which augments the aggregation process. This facilitates the hydrophobic interactions and subsequent aggregation of the central domain of the protein (Moriarty et al., 2017). These results have also been confirmed by single molecule fluorescence studies which has revealed that a slight excess of protons in the solution is conducive towards aggregation of monomeric α-synuclein (Trexler and Rhoades, 2010). However, at a pH below 6, secondary nucleation is favoured than fibril growth thus shifting the kinetics of fibrillation towards formation of toxic protofibrils (Buell et al., 2014). A more recent dissection of the effect of pH on fibrillation has demonstrated that due to the hyperdynamic nature of α-synuclein surface topology, multiple polymorphs of α-synuclein result, each with a different propensity of fibrillation (Frey et al., 2024). The role of the side chain charges in governing the pH susceptibility has also been demonstrated by β-synuclein which develops fibrils at pH 5.8 but not at pH 7.3. As stated in the earlier section, unlike α-synuclein, β-synuclein has a shorter NAC region to readily undergo fibrillation. It was initially thought that the presence of a particular histidine residue at the 63rd position with a pK_a_ of around 6.4 probably serves a critical role in mediating this pH dependent switch of the protein. However, substitution of this histidine with an aspartate residue did not alter the mutant β-synuclein’s behaviour as compared to the wild type with respect to this pH dependent conformational change (Moriarty et al., 2017). Therefore, a more plausible explanation is that at pH 5.8, there are probably enough protons in the solution to shield the significant number of negative charges at the C terminus; however, at pH 7.3, the proton concentration in the physicochemical microenvironment probably falls short to negate these charges. In a LLPS experiment carried out at pH 7.4, α-synuclein in presence of equimolar concentrations of β-synuclein showed a significant increase in turbidity as compared to α-synuclein alone; the corresponding sedimentation experiment indicated that the α/β condensate precipitated readily whereas the β -synuclein was left out in the supernatant justifying β-synuclein’s inability to aggregate at neutral pH (Li et al., 2024).

### 2.5 Effect of phosphorylation

α-synuclein recovered from Lewy bodies of patients affected with sporadic Parkinson’s disease and LBD as well as from glial cytoplasmic inclusion bodies in oligodendrocytes of patients affected with Multiple System Atrophy has been found to be heavily phosphorylated in the 129th serine residue of the C terminal domain (Ghanem et al., 2022). In contrast, in the brains of healthy adults, only 4% of α-synuclein is constitutively phosphorylated (Fujiwara et al., 2002; Hasegawa et al., 2002). Another specific phosphorylation event at S87 found in the brain of patients with synucleinopathies, and the only one at the NAC region has been found to decrease the protein’s membrane binding affinity and also reduces its fibrillation potential (Paleologou et al., 2010). Several important cellular kinases including the G-protein-coupled receptor kinases (GRKs) (Pronin et al., 2000), the casein kinase II (Sano et al., 2021), polo-like kinases (Inglis et al., 2009), and the leucine-rich repeat kinase 2 (LRRK2) (Greggio et al., 2011) mediate phosphorylation of α-synuclein. This suggests a significant role of phosphorylation in regulating fibril formation of α-synuclein. However, contrasting evidences suggest that the timing of phosphorylation is crucial in governing both the initiation nucleation and fibrillation process. Ghanem and coworkers found that efficiency of phosphorylation at S129 increases manifold when protein aggregation has already been initiated (Ghanem et al., 2022). Using state-of-the-art real time quaking induced conversion (Atarashi et al., 2011) of brain homogenates of PD and DLB patients, the authors found that S129 phosphorylation effectively inhibited subsequent fibril formation as well as toxicity associated with α-synuclein. Therefore, it may be safely said that althoughphosphorylation has been demonstrated to augment the fibrillation process; when it occurs at S129 after initiation of the protein fibrillation, both the aggregation propensity as well as resultant cytotoxicity is decreased (Ghanem et al., 2022). A recent MD simulation study has revealed that phosphorylation brings down the average Solvent Accessible Surface Area of the protein thus promoting its hydrophobic core mediated aggregation (de Bruyn et al., 2024).

### 2.6 Effect of glycation

The process of attachment of sugar molecules on lysine and arginine residues of proteins via formation of Schiff’s base is referred to as glycation. It occurs obligatorily due to the abundance of carbohydrates as metabolic intermediates; the accumulation of sugar molecules in specific physiological milieus such as the serum, observed in case of diabetic and prediabetic individuals leads to the formation of non-native protein-sugar adducts more popularly known as Advanced Glycated End Products (Rungratanawanich et al., 2021). Many of the AGE adducts of proteins which are apparently non-amyloidogenic, such as serum albumins, show amyloid like properties (Das et al., 2020; Prosad Banik, 2023). Glycation of α-synuclein is mostly concentrated in the N terminus due to the abundance of lysine residues and affects its membrane binding, thus leading to accumulation of toxic oligomers (Vicente Miranda et al., 2017). Additionally, glycation also interferes with the growing end of α-synuclein fibrillation but does not affect the nucleation step (Farzadfard et al., 2022b). Therefore, it can have a toxic effect on α-synuclein fibrillation by increasing the number of oligomeric species and protofibrils rather than full length fibrils (Kumari et al., 2022). This unique behaviour of glycated proteins also explains the strong clinical correlation between chronic hyperglycemia and susceptibility to neurodegenerative disorders.

### 2.7 Effect of metal ions and their salts on fibrillation

Brain regions affected by neurodegenerative diseases such as PD, Alzheimer’s disease (AD), Amyotrophic Lateral Sclerosis (ALS) and Mass Spectrometry (MS) are found to contain significantly higher concentrations of metal ions such as zinc, iron and copper (Gardner et al., 2017; Miller et al., 2006). The coincidence of cognitive ailments with occupational exposure to these metal ions have been noted on several occasions suggesting a definitive role of the metal ions in disease pathology (Gorell et al., 1997).

α-synuclein fibrillation is strongly affected by metal ions like zinc, copper, iron and calcium (Atarod et al., 2022; Chen et al., 2019). Interestingly, the effect has been significant only in case of divalent/trivalent metal cations (Uversky et al., 2001). Other observations of a high Ferric/ferrous ratio (Zhao et al., 2023) and abundance of oxidised glutathione (Paik et al., 2003) at the SN microenvironment also support the hypothesis that α-synuclein fibrillation is conducive under oxidising conditions. Ferric ions have been shown to bind to a conserved His50 residue in molar equivalents which provides the coordination site for the metal ion. Bound ferric ion decreases lag time and increases rate of fibril formation. However, as reiterated many times in this review, the mechanism of α-synuclein fibrillation is as complex as it can get. Abundance of ferric ions actually halts fibrillation, by inducing formation of spherical oligomers instead of fibrils (Zhao et al., 2023). Probably, the most intriguing effect of metal ions on fibrillation of α-synuclein has been observed with respect to zinc. Unlike the effect of iron, zinc ions are generally known to augment fibrillation. Accumulation of zinc in intracellular milieu has been found to be associated with the death of dopaminergic neurons whereas chelation of zinc reverses the effect (Tamano et al., 2019). However, low concentrations of zinc are actually required by other interacting proteins of α-synuclein to prevent fibrillation (Al-Harthi et al., 2022).

## 3 Role of zinc in modulation of α-synuclein fibrillation

### 3.1 Importance of zinc as a micronutrient in the body

In nutrition, any chemical compound that is required in minute quantity (ranges between 1 and 100 mg/day in adults or constitutes 0%–1% of total body weight) is considered as a “trace element” (Stjernswärd et al., 1996). Zinc is an essential trace element which makes up almost 1.5–2.5 gm of human body weight. Following absorption in small intestine, dietary zinc remains stored in skeletal muscles (60%), bones (30%), skin and liver (5%) and the rest is distributed in different organs like brain, heart, pancreas, and kidney or in mammary glands through blood circulation. In blood, zinc either remains bound with albumins which is exchangeable (75%–85%) or with α-2-macroglobulin which is the non-exchangeable form of serum zinc (15%–20%). Urinary or gastrointestinal excretion of zinc occurs depending on its status in body (Willekens and Runnels, 2022). Zinc, the second most copious trace element after iron in humans has crucial structural, catalytic and regulatory roles as it serves as vital component of more than 2,500 proteins encompassing transcriptions factors and over 100 of enzymes (Kiouri et al., 2023). The spectrum of physiological intervention of zinc is as broad as it gets and includes gene expression and regulation, cell signalling, cell proliferation, apoptosis, DNA metabolism and structural stabilization of chromatin (like sperm chromatin and its decondensation as needed), immune responses, defensive action against oxidants and many other vital physiological processes for sustenance of body homeostasis (Björndahl and Kvist, 2014; Costa et al., 2023) (Table 1).

**TABLE 1:** The diverse physiological roles of Zinc.

Physiological processes	Role/Action	References
Chronic kidney Disease	Antioxidant and anti-inflammatory agents • Cofactor of **superoxide dismutase** enzyme that neutralizes free radicals and reduces oxidative stress in kidney tissues. • Inhibits NF-κB activation, which reduces secretion of pro-inflammatory cytokines (TNF-α, IL-6, IL-1β) that contribute to kidney inflammation. • Prevents lipid oxidation in kidney cell membranes and reducing tissue damage. Kidney fibrosis prevention • Reduces the risk of fibrosis by regulating fibroblast growth factor and prevents excess extracellular matrix deposition. • Reduces proteinuria • Stabilizes integrity of glomerular filtration barrier and reducing protein leakage in urine. • Regulates podocyte function, reduces the risk of proteinuria by preventing kidney cell damage.	Tokuyama et al. (2021)
Immune function	Innate immunity • Maintaining epithelial cell barrier • Activating macrophages and neutrophil • Enhances natural killer cell cytotoxicity • Reduces inflammatory response by cytokine production Adaptive immunity • T cell maturation in the thymus • B cell maturation and antibody production Antiviral and antibacterial action • Inhibits RNA virus replication, like influenza and coronaviruses. • It also prevents bacterial adhesion and reduce infections.	Skrajnowska & Bobrowska-Korczak (2019), Wessels et al. (2017)
Growth and development	• Essential for **DNA replication, RNA transcription, and translation process**, which are essential for cell growth. • Helps in bone matrix formation and mineralization; deficiency can lead to stunted growth and delayed skeletal maturation. • Helps in T-cell and B cell formation, which reduces the frequency of infection and also prevent growth retardation. • Influences the function of **growth hormone and insulin-like growth factor (IGF-1)** **,** both essential for normal growth and development.	(Salgueiro et al., 2002)
Skin health	• Essential for tissue repair and regeneration and helps in collagen synthesis. • Has anti-inflammatory property which reduces redness and irritation of the skin. • Reduces premature ageing and oxidative stress by neutralization of free radicals.	Schwartz et al. (2005)
Reproductive function	Male reproductive system • Essential for spermatogenesis and improve sperm count, motility, and morphology. • Plays a key role in maintaining healthy testosterone levels. • The prostate gland has high concentrations of zinc, which helps to prevent infections. • Necessary for healthy sperm development and genetic material integrity. Female reproductive system • Regulates ovulation process. It helps in oestrogen and progesterone production. • Vital for foetal growth, cell division, and proper development of the baby’s organs. • Plays a role in reducing pre-menstrual syndrome and maintaining a regular menstrual cycle.	Garner et al. (2021), Vickram et al. (2021)
Gastrointestinal function	Supports Gut Barrier Integrity • Essential for maintaining the integrity of the intestinal lining. It strengthens tight junctions between epithelial cells and prevent leaky gut syndrome. Aids Digestion and Nutrient Absorption • Required for the function of carbohydrate and protein digestive enzymes. It supports the production of HCl also. Regulates Gut Immune Function • Helps in the function of immune cells like macrophages and T cells in the intestinal mucosa which has a role in modulating immune responses in the gut and control inflammation Promotes Wound Healing in the Gut • Helps in the repairing of gut lining damage caused by inflammation, infections, or conditions like inflammatory bowel disease (IBD). • Zinc supplementation has been found beneficial in conditions like ulcers and Crohn’s disease. Influences Gut Microbiota • Zinc deficiency can disrupt the balance of gut microbiota, leading to dysbiosis. Prevents and Treats Diarrhoea • Zinc supplementation is widely used to reduce the severity and duration of diarrhoea, especially in children. It also maintains electrolyte balance and supports mucosal immunity. Protects Against GI Infections • Plays an antimicrobial role. It helps to combat pathogens like Helicobacter pylori, which is linked to stomach ulcers	Roohani et al. (2013), Skrovanek et al. (2014)

### 3.2 Role of zinc in minimising ROS and maintaining cellular homeostasis

Zinc serves as co-factor for enzymes involved in defence against oxidative stress. This micro element hinders the activity of pro-oxidant NADPH oxidase, thus maintaining structural stability of the lipid bilayer (Li et al., 2016). Metallothionein (MT) is a cysteine rich protein in cell, which protects from zinc induced toxicity by sequestering the metal. Metallothionine-zinc complex has ROS sequestering activity as it captures and neutralizes the free radicals using cysteine-sulphur ligands; along with its anti-inflammatory and neuroprotective effects (Rodríguez-Menéndez et al., 2018), zinc is also a structural component of the enzyme superoxide dismutase (SOD), found in abundance in the cytoplasm and also a significant enzyme in endogenous antioxidant machineries. SOD contains both copper and zinc and helps to neutralize superoxide radicals thus bringing down the toxicity caused by ROS (Cruz and Soares, 2011). Zinc salts have been also found to be effective in offering protection against lipid peroxidation (Bagchi et al., 1998) by quenching ROS (Bagchi et al., 1997) and also regulates the expression of another cellular antioxidant defence, i.e., glutamate and cysteine ligase, which plays pivotal role in *de novo* synthesis of glutathione. Therefore, zinc can participate directly in ROS scavenging by glutathione or indirectly by acting as cofactor for the enzyme glutathione peroxidise. Ha et al. has shown that concentration of cellular glutathione can be upregulated by *in vitro* administration of regulated amount of zinc in epithelial pigment cells of human retina (Tan et al., 2013). Metal Transcription Factor-1 (MTF-1) is dependent on zinc. When the zinc concentration in body rises, it expresses gene for metallothionine and zinc transporter-1 and maintains zinc homeostasis and avert excess metal induced oxidative stress (Günther et al., 2012). MTF-1 also promotes the expression of selenoprotein-1 which binds glutathione and takes part in ROS scavenging. The inertness of zinc towards catalysis of free radical generation reactions additionally boosts its antioxidative efficacy (Oteiza, 2012). Zinc can bind with either thiol or a sulfhydryl group in proteins thus hindering the intra-molecular formation of disulfide (Choi et al., 2018). Zinc alleviates oxidative stress as an anti-inflammatory element by taking part in various molecular signalling pathways which increases the expression of anti-inflammatory protein A20 (otherwise known as TNFAIP3) and downregulates the expression of the pro-inflammatory NF-kB (nuclear factor κB) (Prasad et al., 2010). Zinc has also shown potent effect in attenuation of pro-inflammatory cytokines including Interleukin-6, Tumour Necrosis Factor α (TNF-α), and C-Reactive protein and stimulation of the molecular signalling pathway leading to expression of the A20 protein (Marreiro et al., 2017). Zinc also upregulates the expression of Nrf2, a chief mediator of antioxidant machinery (Olechnowicz et al., 2018).

Zinc has a crucial role in the maintenance of intracellular calcium concentration. Its deficiency causes calcium build-up promoting the release of substance P, a neuropeptide involved in immune response and inflammation resulting in oxidative stress (Weglicki et al., 2011). Deficiency of this micro-element also leads to activation of NADPH oxidase, ROS and reactive nitrogen species (Lee et al., 2021). As zinc is involved in synthesis of the insulin along with its storage and release, it plays a significant role in blood glucose homeostasis with implications in type 2 diabetes, obesity, cardiovascular diseases, altered lipid metabolism and metabolic syndromes (Motamed et al., 2013).

The role of zinc is integral in neuronal health, especially for carrying out routine physiological processes of the Central Nervous System (Li et al., 2023) (Table 2). The hippocampus, amygdala and cortex are the major storage sites of the zinc and a subtly regulated flux of zinc from these regions are vital for carrying out critical functions of the brain including cognition, memory and emotional stability. Scientific evidences have unanimously established the role of zinc ions in modulating glutaminergic synapse transmission (Bertoni-Freddari et al., 2008). The same has been extensively reviewed by Sikora et al. (2020). Physiological concentrations of zinc range between 10 and 50 µM in and around the vicinity of the Substantia nigra (SN) which is typically much higher than that required elsewhere in the body and justifies the diverse integral roles of the metal in the brain. Further rise in concentration of the metal is often met with severe cognitive ailments (Sabouri et al., 2024; Sandstead et al., 2000). Therefore, it is equally essential to control the level of zinc in these cellular micro-niches. ATP13A2 is a P type ATPase generally found in the membranes of acidic vesicles in neurons which, along with the other two more common class of Zinc transporters belonging to the ZnT (SLC30) and ZIP (SLC39) families, is entrusted to serve exactly this role (van Veen et al., 2014). Overexpression of a ATP13A2 orthologue in yeast was shown to impart zinc resistance (Kong et al., 2014); on a different note, an individual with a mutant of ATP13A2 was affected with Kufor-Rakeb syndrome, a disease with shared features of Parkinsonism (van Veen et al., 2014). These observations were subsequently confirmed in a mouse PD model where zinc was shown to increase α-synuclein aggregation; overexpression of ATP13A2 neutralized the effect of zinc thus alleviating α-synuclein pathology whereas its knockout significantly aggravated fibrillation (Gao et al., 2022). It has been confirmed using many independent approaches that the presence of zinc results in increased content of beta structure of α-synuclein.

**TABLE 2:** Role of zinc in nervous system and cognitive development.

Physiological processes	Role/Action	References
Brain development	• Involved in neurogenesis (and synaptogenesis). • Helps in cell division and differentiation, which are crucial for brain structure and function. • Present in high quantity in the hippocampus region of brain which is responsible for learning and memory	Chudasama et al. (2008), Sensi et al. (2018)
Neurotransmission and Synaptic Plasticity	• Modulates the activity of neurotransmitters such as glutamate, dopamine, and serotonin, which are essential for crucial changes of mood, cognition, and learning. • Enhances synaptic plasticity and key factor for memory formation	Krall et al. (2021)
Cognitive Functions	• Essential for cognitive function, including problem-solving, reasoning, and also improve level of concentration. • Supports long-term potentiation, which is essential for learning and memory storage	Li et al. (2023)
Mood Regulation and Mental Health	• Deficiency is linked to depression, anxiety, and attention-deficit hyperactive disorders. • Regulates cortisol levels and preventing excessive stress-related brain damage. • It controls neurotransmitters dopamine and serotonin which is associated with mood and stability.	Sensi et al. (2018)
Protection Against Neurodegeneration	• Has antioxidant and anti-inflammatory properties, protecting neurons from oxidative stress and inflammation. • By preventing protein aggregation and neuronal damage reduces the risk of neurodegenerative diseases like Alzheimer’s and Parkinson’s	Xie et al. (2020)
Role in Brain Development During Pregnancy and Childhood	• During foetal development, zinc is crucial for brain formation, nerve growth, and early cognitive function. • Deficiency during pregnancy can lead to congenital brain defects and impaired intellectual development in infants.	Goeldner et al. (2008)
Neuroprotection and Antioxidant Activity	• Acts as an antioxidant, reducing oxidative stress and neurodegenerative diseases like Alzheimer’s and Parkinson’s where neuron damage occurs. • It helps in maintaining the integrity of the blood-brain barrier, and preventing harmful substances entering into the brain	Xie et al. (2020)
Prevents Neurodegenerative Diseases	• Reduces amyloid-beta plaque formation, which is linked to Alzheimer’s disease. • Prevents excessive glutamate excitotoxicity, which can lead to neuronal damage	Xu et al. (2019)
Regulates Sleep and wake cycle	• Influences melatonin production, which helps regulate the sleep-wake cycle	Cherasse and Urade (2017)
Supports Nervous System Repair	• Aids in nerve regeneration and repair after injury. • Plays a role in maintaining myelin sheaths formation, which protect nerve fibres and facilitate efficient nerve signalling	Kumar et al. (2021)

In contrast to toxic effects of zinc reported by most studies, the metal has been shown to mitigate oxidative stress (Méndez-Álvarez et al., 2002) as well as prevent toxicity of dopaminergic neurons (Ajjimaporn et al., 2008). The physiological role and requirement of zinc is evident from the fact that zinc is stored in within synaptic vesicles at the terminals of glutaminergic neurons especially in the forebrain region. It is released along with glutamate and plays an integral role in mediating transmission of synapse through NMDA and GABA receptors (Anderson et al., 2017; Vergnano et al., 2014). Pathological consequences start occurring when an excess of synaptic zinc leaks in to the post synaptic neurons through the ion channels (Sensi et al., 2011).

### 3.3 Understanding the basis of the “zinc effect” on α-synuclein

As stated previously, many divalent metal cations have the potential to augment the fibrillation of α-synuclein by a common mechanism of shielding the negative charge density on the protein. The effect is more prominent in case of the trivalent lanthanides which can additionally bind to the central NAC region and accelerate fibril formation (Bai et al., 2016). A closer look reveals that different cations vary with regards to their binding affinity to α-synuclein and subsequent effect on the kinetics of nucleation and elongation.

**FIGURE 3:**
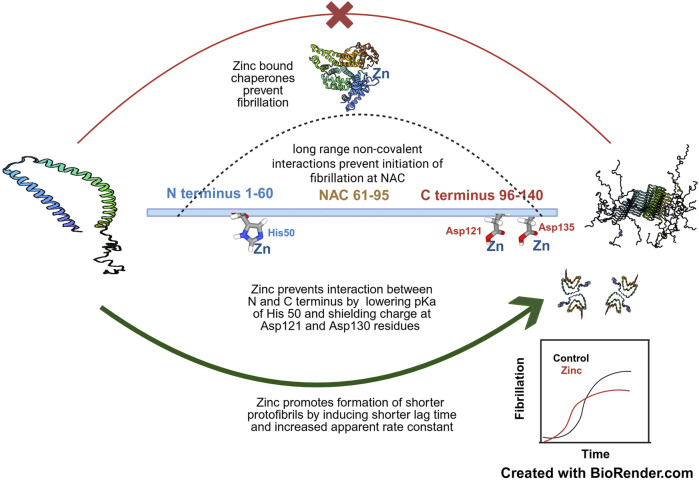
The concentration dependent effect of zinc on α-synuclein fibrillation- Zinc at moderate to high concentrations (around 100 µM) promotes aggregation of NAC domain by shielding the C terminal negative charges of Asp121 and Asp135 and at even higher concentrations also lowers the pKa of His50 at the N terminus-the cumulative effect is to prevent the long range non-covalent interaction of the N and C terminus which prevents initiation of fibrillation. Zinc induces formation of short fibrils by decreasing lag time and increasing association constant as represented in the inset. However, lower concentrations of zinc helps to prevent fibrillation by assisting in the chaperonin activity of proteins which keeps α-synuclein in its physiological conformation. The PDB files of α-synuclein, namely, 1XQ8 (Ulmer et al. 2005), 2N0A (Tuttle et al. 2016) and 6OSM (Ni et al. 2019) and HSA, 6YG9 (Mishra et al. 2020) have been used in this figure. The concepts and mechanisms depicted in the figure have been adopted from multiple studies as cited in the text. Figure has been created with BioRender.

Native nano-electrospray ionisation and ion mobility-mass spectrometry (nESI(-IM)-MS) can differentiate conformations of the protein based on their charged states, relying on the principle that denatured proteins are more extended and therefore are comparatively more protonated than native compact states with a lower Solvent Accessible Surface Area. Therefore, denatured conformations of proteins can readily be differentiated from the native conformers based on their status of protonation. Due to the inherently Intrinsically Disordered nature of α-synuclein, an ensemble of native state monomers of the proteins can be distinguished with a charge ranging between +5 and +18. Using this technique, the binding of zinc to the protein was probed quantitatively and at equimolar ratios of metal ion: protein, zinc was found to exhibit significant binding to α-synuclein along with sodium, copper and lanthanum (Moons et al., 2020). In a subsequent study conducted to understand the effect of zinc on α-synuclein fibrillation, the metal ion was found to decrease the lag time of aggregation (t_lag_) with a simultaneous increase in apparent rate constant (k_app_). However, the fibrils formed were significantly shorter than those formed in the presence of copper, calcium and magnesium (Atarod et al., 2022). FTIR analysis of amide I vibrational spectrum also confirmed that in presence zinc, additional regions of the protein take up the beta pleated sheet conformation (Li et al., 2022). A combination of single residue resolution level NMR spectroscopy and truncated versions of α-synuclein mutants have revealed the binding sites of zinc. In presence of 100 µM zinc, the most significant binding site has been deciphered to be the Asp121 and Asp135 in the C terminus; however, no additional binding interaction was noted due to the presence of other negatively charged residues in the vicinity including Glu130, Glu131, Glu137 and Glu139. This clearly indicated that the affinity of zinc for the C terminal domain was not solely governed by only electrostatic interactions but was primarily a result of the unique conformation of the protein centred around the Asp121 residue. Binding at a threefold excess molar concentration of zinc revealed the second binding site of the metal at His50 in the N terminus but no additional affinity was noted for the C terminus. The trend of zinc binding to α-synuclein is in unison with the observed effects; at low physiological concentrations, zinc serves a twofold function of both neutralizing the negative charge density at the C terminal of α-synuclein and assists other chaperones which work in a concerted manner to prevent aggregation of α-synuclein, whereas at higher concentrations, zinc promotes fibrillation by binding to His50 at the N terminus and lowering its pK_a_, much like that observed in case of iron (Valiente-Gabioud et al., 2012). Given the fact that physiological concentrations of zinc seldom exceed 50 μM at the substantia nigra micromilieu, it is apparent that *in vivo*, zinc induced α-synuclein toxicity is not an issue of concern. Interestingly enough, the shorter fibrils formed in presence of zinc were not cytotoxic unlike the longer ones formed in presence of the other cations (Atarod et al., 2022). This observation came as a surprise given the fact that oligomers of α-synuclein impart more toxic effects than the fully mature fibrils due to their stronger ability to induce pore formation in the membrane (Zhang et al., 2013). One of the several possible answers to explain this may lie in the fact that zinc ions serve as metal activators of certain chaperonin proteins which bind and neutralize synuclein oligomers (Al-Harthi et al., 2022). Another clue to explain the apparent harmlessness of zinc induced synuclein oligomers comes from a related study in tau proteins implicated in disease pathology of AD. Zinc induced oligomerization of tau occurred in a temperature dependent manner without the necessity of additional cofactors such as heparin. However, these oligomers did not eventually develop into amyloid fibrils and were dissociated on lowering the temperature (Roman et al., 2019) indicating the reversibility in the formation of the oligomers. The results if extrapolatable in case of α-synuclein will imply that oligomers of α-synuclein formed in the presence of zinc are also transient unstable entities not capable of inducing pores in lipid membranes. A similar study conducted by Kim et al. much earlier, had indicated that heat induced aggregation of recombinant α-synuclein was facilitated in presence of zinc (Kim et al., 2000). A possible explanation of these findings is the fact that increasing temperature augments hydrophobic interaction at the NAC region of α-synuclein; however, at the same time temperature lowers the pKa of the acidic side chains in the C terminus thus facilitating the opposite effect of keeping fibrillation at bay. Zinc masks these negative charges even at elevated temperatures to promote fibrillation. As soon as the temperature drops, the hydrophobicity driven factor is neutralized, rendering the proteins into random aggregates which lack a structured fibrillar component and therefore incapable of effecting membrane damage. Zinc is also known to induce liquid-liquid phase separation of proteins implicated in neurodegenerative disorders (Singh et al., 2020; Yatoui et al., 2022). The metal was shown to induce phase separation in tau, the amyloid like protein associated with AD pathology and shift the equilibrium phase boundary of LLPS towards the region of lower protein concentration (Singh et al., 2020). However, there has been little or no study on the effect of zinc in the LLPS of α-synuclein. The dual role of zinc on aggregation and fibrillation of α-synuclein has been summarised in Figure 3.

### 3.4 Role of other micronutrients in shaping amyloid fibrillation: iodine and selenium in regulating α-synuclein fibrillation

Apart from zinc, other physiologically significant non-metal micronutrients such as iodine and selenium have also been demonstrated to possess anti-amyloidogenic properties. Iodine is an acclaimed chaotropic agent capable of modulating the conformational stability of a plethora of proteins by reorienting the water structure around the protein’s microenvironment. Iodine has also been shown to interact with amyloid fibrils, amyloid polymorphs and form complex with mature insulin amyloid fibrils (Hiramatsu et al., 2020; Takekiyo et al., 2022). By using a combination of biophysical techniques, Taketiyo et al. demonstrated that ethylammonium iodide (EAI) can inhibit the formation of α-synuclein amyloid aggregate via interaction between I_3_^−^ ions and Lys residues in the N-terminal domain of the protein (Takekiyo et al., 2022).

Selenium and selenoproteins have also been implicated in modulating α-synuclein fibrillation. Free Selenium can interact with α-Synuclein and induce the formation of protein aggregates (Maass et al., 2020). SelS and SelP are the two major selenoproteins of the body implicated in antioxidant defence and selenium transport respectively (Lu and Holmgren, 2009). Additionally, SelS has been described as the regulator of the protein aggregation; it probably acts by mediating transcriptional downregulation of aggregation prone proteins; however, the exact mechanism is yet to be deciphered. A recent study on PD patients has suggested that serum concentrations of Se and SelP can potentially be used as a biomarker for monitoring α-Synuclein aggregation (Salaramoli et al., 2024).

### 3.5 Leads towards zinc based therapeutic strategies to counteract synucleinopathies

There are currently very few safe therapeutic strategies to directly degrade and clear amyloid fibrils. One of these is the bacterial enzyme nattokinase which is a serine protease capable of efficiently removing fibrinolytic clots (Ni et al., 2023). A new enzyme from earthworm termed lumbrokinase has been reported by Girigoswami et al. with the potential to degrade fibrin clots (Metkar et al., 2017). The polysaccharide carrageenan isolated from certain species of red algae possess the potential to degrade amyloid fibrils (Makshakova et al., 2023). Girigoswami et al. has recently developed a nanoformulated carrageenan with enhanced degradation potential as compared to the conventional one (Udayakumar et al., 2024).

The ability of zinc to arrest fibrillation by assisting chaperones and to induce formation of non-toxic protofibrils and oligomers have led to the prospects for the development of zinc oxide nanoparticle-based biomarkers. In a study carried out by Asthana et al., zinc oxide nanoparticles showed significant binding affinity with α-synuclein fibrils via a) enthalpy driven interaction with the amphipathic “KA: TKE/QGV” repeats and b) enthalpy-driven interaction with the central NAC region as well as the C terminus; this led to arrest of subsequent fibrillation (Asthana et al., 2020). Based on this observation, a zinc oxide nanoparticle coated aluminium microelectrode has been designed as an efficient tool for early detection of α-synuclein fibrillation (Adam et al., 2021a). A related study based on Surface Enhanced Raman Spectroscopy reported that zinc oxide nanoparticles can effectively bind and sequester α-synuclein oligomers provided they have not been exposed to physiological temperatures (37°C) which would lead to irreversible formation of amyloid fibrils (Slekiene et al., 2022). Further refinement in this regard has been achieved with surface moderated zinc oxide nanoparticle corona which is able to effectively remove flocs or amorphous aggregates of α-synuclein (Jena et al., 2025). In order to maximize the surface area for enhanced sensitivity in detection of amyloids, a zinc nanoflower-based on nano-silver thin film has been developed (Akhtar et al., 2017) and subsequently upgraded to degrade the amyloid fibrils (Girigoswami et al., 2019). As discussed before, Thioflavin-T positive glycated proteins in the bloodstream of diabetic or prediabetic patients can elicit amyloid like complicacies. Similar studies exploring the effect of zinc nanoparticles on glycated proteins need to be conducted to determine whether zinc-oxide nanoparticles can also trap these protein-sugar adducts before they develop into toxic amyloids capable of developing cognitive ailments and other complicacies.

## 4 Discussion: bottlenecks in current technology and future of research in alpha-synucleinopathies

The discovery of the strong clinical correlation between Type 2 diabetes and neurodegeneration and the molecular crosstalks involved therein has opened up new avenues of research in elucidating the molecular and physiological basis of cognitive decline, but at the same time it has also seriously complicated our understanding of complex neurodegenerative diseases such as PD and other alpha-synucleinopathies. As already stated, serum proteins existing in a high sugar milieu for a long period develop into Advanced Glycated End products (AGEs) via Amadori reaction and subsequent Maillard rearrangement. Many of these adducts have shown Thioflavin-T positive amyloidogenic behaviour (Hsu et al., 2019). Several other proteins irrespective of the state of glycation, show resemblance to amyloids and collectively constitute the “amyloid proteome” (Gottwald and Röcken, 2021). Although the nature and fibril forming mechanism of most of these proteins have been delineated in detail, the same is not true for the glycated adducts. The burden of glycated proteins can be estimated from the fact that diabetic individuals are more than 50% susceptible in developing cognitive disorders as compared to non-diabetic persons (Cao et al., 2024). The management of glycated proteins is further escalated by the fact that they are recalcitrant to ubiquitin mediated degradation owing to selective modification of lysine residues via glycation. Therefore, efficient and early detection of glycation is pivotal for avoiding many diseases.

**FIGURE 4:**
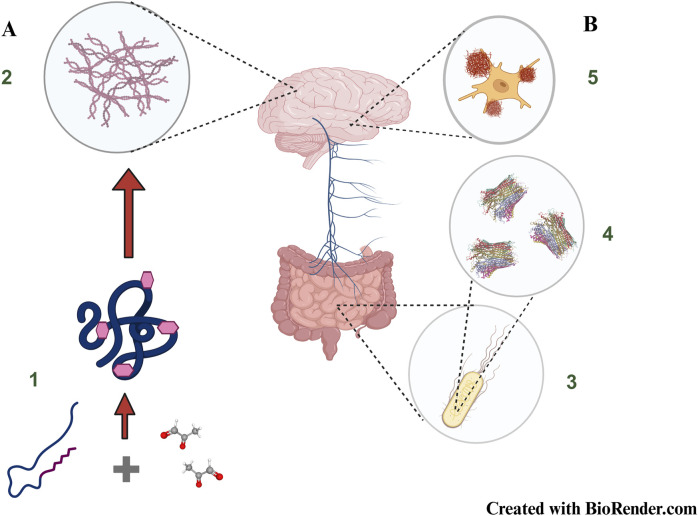
New frontiers in brain amyloid formation: **(A)** Non-enzymatic glycation of proteins resulting in the formation of Advanced Glycation End Products (1) mimic amyloid like behaviour (2) and constitute one of the major causes to account for diabetes induced neurodegeneration **(B)** Gut resident *Escherichia coli* (3) produce bacterial amyloids known as curli (4) which can potentially be transported to the brain via the vagus nerve (Gut-Brain Axis) and influence abnormal behaviour of resident amyloidogenic proteins (5). The cartoon structure of Curli has been taken from PDB ID 6LQH, Zhang et al. 2020.The concepts and mechanisms depicted in the figure have been adopted from multiple studies as cited in the text. Figure has been created with BioRender.

Previously, people used immunological techniques such as ELISA and immunoblotting for detection of glycated protein adducts which frequently led to overestimation of AGEs due to conversion of Amadori products into carboxy methyl lysine or pentosidines (Suzuki et al., 2022). Thereafter, improvisations on fluorescence-based methods such as measurement of skin autofluorescence to detect skin AGEs (Koetsier et al., 2010) has facilitated detection of subcutaneous deposition of AGEs. In spite of the intrinsically challenging task of ionisation of glycated proteins and peptides owing to their charged status (Bae et al., 2012), detection of glycation has also been made possible by the development of advanced proteomic tools like stable isotopic dilution analysis liquid chromatography-tandem mass spectrometry and stable isotope labelling with amino acids in cell culture (SILAC) high resolution mass spectrometry (Rabbani et al., 2016). It must, however, be reinstated that our understanding of the role of glycation in mediating neurodegenerative disorders is still at rudimentary level, and preliminary studies have indicated that although metal ions like zinc and iron inhibit glycation of amyloidogenic proteins (Baraka-Vidot et al., 2014). Another potential but less explored avenue on amyloid plaque formation research is the role of bacterial Curli system on cellular amyloid proteins such as α-synuclein and amyloid protein A. Curli is itself an amyloid like protein and an integral component of biofilm produced by some strains of *Escherichia coli* and several *Salmonella* sp. (Oh et al., 2012). Inside human body, curli, produced by resident *E. coli* of gut can be transported to the brain via the Gut-Brain axis (Zheng et al., 2023). Curli can both augment or inhibit the fibrillation of human amyloids depending on the type of protein and/or cellular conditions (Christensen et al., 2019; Ivanova et al., 2021). Zinc is an essential constituent of Curli biogenesis in *E. coli* and while in gut, the bacterium draws its requisite zinc from the physiological reserve. YkgM and ZinT proteins maintains the optimal zinc concentration required for Curli biogenesis and assembly (Lim et al., 2011). However, it is imperative to state that more oriented studies need to be conducted to understand the role of zinc and other metals in mediating the fibrillation microenvironment of bacterial curl as well as other its effect on other human amyloids. A schematic representation of amyloids resulting from glycated adducts as well as the mode of transport of *E. coli* curli via the vagus nerve to the brain is presented in Figure 4.

## 5 Conclusion

Management of α-synucleinopathies has been an insurmountable hurdle for both the scientific as well as the biomedical community. The delineation of almost all structural and physicochemical facets of α-synuclein has facilitated the development of biomarkers to detect protein aggregates associated with cognitive ailments such as synucleinopathies (Bagree et al., 2023). Particularly, significant progress has been made possible through the use of Surface Enhanced Raman Spectroscopy (SERS), a technique which achieves detection of miniscule analytes through amplification of Raman scattering and thus can diagnose a cognitive problem before the actual onset of the symptoms (Colniță et al., 2023). However, we are yet to reach the desirable Technology Readiness Level to adopt these tools in a real-life clinical setting (Ganguly et al., 2021). In this regard, the role of zinc has emerged on a dual perspective in both prevention and cure of these complex multifactorial ailments. The intrinsically strong zinc-α-synuclein interaction has serious prospects for circumventing the current technological bottlenecks. A gold nano-rod incorporated zinc-oxide nanocomposite has stepped a foot forward by discriminating between native and fibrillated α-synuclein (Adam et al., 2021b) and seems to hold a lot of promise in identification of the chemical microenvironment conducive to fibrillation. The high affinity of zinc especially towards the C-terminal acidic domain of α-synuclein has facilitated the development of zinc-oxide nanocomposites to probe the deposition of α-synuclein aggregates (Di Mari et al., 2024). More importantly, considering the fact that many neurodegenerative ailments have been traced to have roots in lifestyle diseases, proper management of our diet including adoption of food processing techniques is of paramount importance to ensure the requisite absorption and bioavailability of micronutrients including zinc.
